# Identifying Early Presentation of Serotonin Syndrome in a 20-Year-Old Man Prescribed Clomipramine and Quetiapine: A Case Report

**DOI:** 10.7759/cureus.92487

**Published:** 2025-09-16

**Authors:** Veronica Alday, Justin Scarano, Jonathan Y Yuh, Christie Richardson

**Affiliations:** 1 Department of Psychiatry, Rowan-Virtua School of Osteopathic Medicine, Mt. Laurel, USA

**Keywords:** clomipramine, critical care psychiatry, hunter criteria, medication overdose, obsessive compulsive disorder, quetiapine, serotonin syndrome, tricyclic antidepressant

## Abstract

Serotonin syndrome can be challenging to diagnose due to its variable clinical presentation and multiple potential drug triggers. This may present from mild to fatal, with symptoms such as autonomic instability, altered mental status, and neuromuscular excitation often manifesting as hyperreflexia and clonus. We present a 20-year-old man with a past medical history of obsessive compulsive disorder (OCD) being treated with clomipramine 25 mg orally (PO) daily and quetiapine 100 mg PO daily, who arrived at the emergency department for agitation. He was found to be in sinus tachycardia with a heart rate (HR) around 140 beats per minute (bpm), with no medical or substance cause identified. He was medically stabilized, continued on his home medications, and transferred to the inpatient psychiatry unit. However, he again became tachycardic at 175 bpm, with muscle rigidity, tremor, agitation, and diaphoresis. The patient’s HR stabilized following administration of lorazepam 2 mg intramuscular (IM) and metoprolol 5 mg intravenous (IV), but he remained rigid and diaphoretic for several more hours. The patient reported that he had taken clomipramine in excess prior to admission, so clomipramine was discontinued prior to discharge. In this case, muscle rigidity masked the hyperreflexia and myoclonus often used to diagnose serotonin syndrome, making it even more difficult to recognize. Additionally, collateral information about the patient’s excessive use of clomipramine, a potent serotonergic tricyclic antidepressant (TCA), was not obtained early on. Few cases of mild and masked serotonin syndrome have been reported due to the difficulty in recognizing and quickly treating such cases. Cases of serotonin syndrome should continue to be reported in order to familiarize clinicians with the varied presentation.

## Introduction

Serotonin syndrome is a potentially life-threatening condition caused by overstimulation of serotonergic receptors and can vary in presentation, severity, and symptomatology. Because not all common symptoms may be present at once, some cases may be missed, impacting patient outcomes and mortality. With the increasing use of serotonergic agents used for anxiety and depression, the incidence of serotonin syndrome has also increased, making the ability to recognize it and evaluate individual risk even more important [1]. 

Early presenting symptoms of serotonin syndrome include hyperreflexia, diarrhea, nausea, and insomnia, but presentation can vary based on the amount of serotonergic activity in the central nervous system and which serotonergic receptor subtypes are active [1,2]. Broadly, serotonin syndrome presents with neuromuscular abnormalities, autonomic hyperactivity, and mental status changes. Because serotonin syndrome often does not produce a clear clinical picture, no universally accepted diagnostic criteria reliably identify all cases. Currently, the Hunter Criteria are used to classify the condition, but mild cases continue to be under-recognized [3].

Risk of serotonin syndrome increases in patients who are taking two or more serotonergic agents, but can also occur in more susceptible patients who are only taking one serotonergic medication [1,4]. Numerous drugs affect serotonergic neurotransmission, including serotonin reuptake inhibitors (SSRIs), antiepileptics, tricyclic antidepressants (TCAs), monoamine oxidase inhibitors (MAOIs), antiemetics, and opioids [3]. Clomipramine is a TCA that is approved for obsessive-compulsive disorder (OCD) and sometimes used off-label for depression; however, it is known to cause serotonin syndrome when used in excess or in overdose [5]. This case report describes a patient who develops masked symptoms of serotonin syndrome after excessive use of clomipramine and exemplifies the importance of identifying early and masked signs of serotonin syndrome.

This article was previously presented as an electronic poster at the 2025 Pennsylvania Psychiatric Society Colloquium of Scholars on March 29, 2025, and the 29th Annual Rowan-Virtua SOM Research Day on May 1, 2025.

## Case presentation

A 20-year-old white man presented to the emergency department with his family for impulsive behaviors. He had a past medical history of depression, generalized anxiety disorder, and OCD, and was being prescribed clomipramine 25 mg orally (PO) daily and quetiapine 100 mg PO daily. The patient’s mother reported that the patient had been noncompliant with his medications over the past six weeks, and she was afraid to leave the patient alone because of his erratic behaviors and worsening paranoia. The patient reported that he had been having difficulty falling and staying asleep due to persistent intrusive thoughts and excessive caffeine intake, amounting to at least 32 ounces of coffee daily. On examination, he was found to have persistent sinus tachycardia with his heart rate ranging from 102 to 127 bpm, body temperature of 37.1°C, blood pressure of 137/81 mmHg, and oxygen saturation of 98%. Complete blood count, complete metabolic panel, calcium, thyroid-stimulating hormone, and D-dimer level were all within normal limits with the exception of mild hypokalemia, which stabilized and responded to 40 mEq of KCl (Table 1). A 2D echocardiogram was normal. Urine drug screen was unremarkable. The patient was also restarted on his home medication of clomipramine 25 mg PO and quetiapine 100 mg PO before being transferred voluntarily to the inpatient psychiatric unit that day.

**Table 1:** Laboratory Values on Initial Presentation

Parameters	Patient values	Reference range
White blood cell count	9.26 × 10^3^/uL	4.80-10.80 × 10^3^/uL
Hemoglobin	14.2 g/dL	13-18 g/dL
Hematocrit	40.80%	40%-52%
Platelets	263 × 10^3^/uL	130-400 × 10^3^/uL
Sodium	141 mmol/L	136-145 mmol/L
Potassium	3.2 mmol/L	3.4-4.8 mmol/L
Chloride	103 mmol/L	98-107 mmol/L
Calcium	9.6 mg/dL	8.6-10.0 mg/dL
Creatinine	0.82 mg/dL	0.67-1.20 mg/dL
Glucose	97 mg/dL	70-100 mg/dL
Thyroid-stimulating hormone	1.310 uIU/mL	0.270-4.200 uIU/mL
D-dimer	<500 ng/mL	<500 ng/mL

In the psychiatry unit, the patient was still demonstrating active paranoia, disorganized thinking, fidgeting, and perseverating on the idea that he was not well understood by others and that people kept “pushing me away.” He did admit to obsessions and compulsive behaviors. He recalled a previous psychiatric hospitalization in 2022, where he was diagnosed with OCD and was prescribed clomipramine during this time. The patient added that since then, he had been trying to wean himself off of his medication and manage his symptoms on his own, but would “catch up” on the medication on other days by taking excessive amounts, which he did admit to doing prior to this hospitalization, but he was unsure of how much he took. He denied suicidal ideation or behaviors, homicidal ideation, or legal troubles. At this time, there was high suspicion of first break psychosis given his age and presentation of symptoms; however, shortly after admission, the patient began to demonstrate muscle rigidity, tremulousness, and diaphoresis, with a heart rate reaching 175 bpm, blood pressure of 147/80 mmHg, and respiratory rate of 24. Rapid response was called, and the patient was given lorazepam 2 mg intramuscular (IM) and metoprolol 5 mg intravenous (IV), which brought his heart rate down to 126 bpm, but he was still experiencing muscle rigidity in all four extremities and diffuse tremor. The patient was transferred to telemetry for further monitoring, and his psychiatric medications were held.

Complete blood count, basic metabolic panel, and metanephrines to rule out pheochromocytoma were within normal limits, with the exception of an elevated cortisol level (Table 2). Endocrinology attributed this to acute stress, as the patient’s dexamethasone suppression test showed appropriate suppression. Therefore, abnormal cortisol overproduction and hypercortisolism, which could present with symptoms similar to serotonin syndrome, were effectively ruled out. The patient’s muscle rigidity, tremors, tachycardia, and diaphoresis resolved within 24 hours, and he was discharged from the medicine floor with 25 mg of metoprolol tartrate to be taken every 12 hours.

**Table 2:** Laboratory Values Upon Transfer to Telemetry Unit

Parameters	Patient values	Reference range
White blood cell count	9.8 × 10^3^/uL	4.80-10.80 × 10^3^/uL
Hemoglobin	13.9 g/dL	13-18 g/dL
Hematocrit	39.90%	40%-52%
Platelets	258 × 10^3^/uL	130-400 × 10^3^/uL
Sodium	141 mmol/L	136-145 mmol/L
Potassium	3.6 mmol/L	3.4-4.8 mmol/L
Chloride	106 mmol/L	98-107 mmol/L
Calcium	9.6 mg/dL	8.6-10.0 mg/dL
Creatinine	0.81 mg/dL	0.67-1.20 mg/dL
Glucose	92 mg/dL	70-100 mg/dL
Metanephrine, plasma	34 pg/mL	0-88 pg/mL
Normetanephrine, plasma	135.5 pg/mL	0.0-210.1 pg/mL
Cortisol	20.7 ug/dL	2.3-19.4 ug/dL

The patient was diagnosed with serotonin syndrome based on his history of clomipramine overuse and the temporal progression of his symptoms. The patient was transferred back to the psychiatric unit, where he expressed that he no longer wanted to take clomipramine. The patient was started on olanzapine 5 mg PO daily for OCD, and clomipramine and quetiapine were not restarted. He was discharged shortly after and was advised to follow up with his outpatient psychiatrist and therapist.

## Discussion

While the risk of serotonin syndrome associated with serotonergic agents is well-known, cases have historically been difficult to diagnose. Serotonin syndrome, particularly mild cases with atypical presentation, can be challenging to recognize and can delay imperative treatment. Accurate diagnosis and comprehensive workup for serotonin syndrome is a necessary skill for all practitioners, especially as more patients are being treated with serotonergic medications. Serotonergic agents are often implicated to treat a number of conditions, including depressive disorders, anxiety disorders, trauma disorders, and OCD. As more patients are prescribed serotonergic agents, the risk of serotonin syndrome is a growing concern, especially with polypharmacy being so prevalent and multiple medications having serotonergic properties that are not recognized.

Serotonin syndrome often presents with the classic triad of altered mental status (which can include anxiety, agitation, and delirium), autonomic instability (including diaphoresis, tachycardia, hyperthermia, hypertension, vomiting, and diarrhea), and neuromuscular excitability (including myoclonus, tremor, hyperreflexia, and muscle rigidity) [1,3]. These symptoms often do not present concomitantly. As the presentation of serotonin syndrome is influenced by the patient’s medications, the specific subtypes of receptors involved, and the degree of serotonin overexcitability, mild cases can be challenging to diagnose and differentiate from other medical conditions, such as neuroleptic malignant syndrome, anticholinergic syndrome, antidepressant discontinuation syndrome, and central nervous system stimulant overdose [2,4]. In the past, the Hunter Criteria were the most widely accepted set of symptomatology used to guide this clinical diagnosis, but mild cases tend not to follow this algorithm and are often overlooked [1,2,6]. A complete history and high clinical suspicion are essential to evaluate a patient with a mild case of serotonin syndrome, as in this patient. Treatment for mild serotonin syndrome involves discontinuing the offending agent, supportive therapy including IV fluids, correction of vital signs, and symptomatic treatment with a benzodiazepine. In more severe cases, advanced interventions such as intubation, paralysis, and sedation may be necessary [4]. Hypertonia is managed with sedation and intubation to alleviate muscle rigidity and support mechanical ventilation. Hyperthermia, which results from excessive muscle activity, rather than a change in hypothalamic set point, should be managed with paralysis and standard cooling measures. Antipyretics have no effect and are not recommended [1,2,4]. Serotonin antagonists such as cyproheptadine and chlorpromazine can also be considered to treat serotonin toxicity; however, their efficacy can vary [1-4]. Cyproheptadine is often preferred due to its potent 5-HT2A receptor antagonist activity. In contrast, chlorpromazine can cause severe orthostatic hypotension and should be avoided in hypotensive patients [1,3,4].

While serotonin syndrome is often associated with SSRIs and MAOIs, TCAs are also associated with an increased risk that is not as well-studied. Clomipramine’s efficacy is undisputed, and it is often prescribed to patients being treated for OCD who cannot tolerate or do not respond to SSRIs. However, clomipramine has been found to have an unfavorable side effect profile, causing higher rates of sedation, dry mouth, nausea, constipation, dizziness, vision changes, headache, and seizures [5]. This may be due to clomipramine’s serotonin reuptake inhibitor properties, which give it a stronger affinity for the serotonin transporter than other drugs in its class, producing a more potent effect on serotonin levels and increasing its efficacy [5,7]. Although clomipramine effectively addresses the serotonergic imbalance involved in the etiology of OCD, it subsequently also increases one’s risk of developing serotonin syndrome. Despite the high risk of serotonin syndrome associated with clomipramine use, few cases associated with this medication have been reported.

This patient’s presentation of serotonin syndrome was atypical, as he was not being medicated with a cocktail of serotonergic medications often associated with the clinical diagnosis. In addition to clomipramine, he was also prescribed quetiapine, a dopamine antagonist and partial agonist at 5-HT1A, which also increased the risk for serotonin syndrome. He first presented with autonomic instability and some level of altered mental status, specifically arriving at the emergency department with paranoia, intrusive thoughts, and tachycardia. However, the patient’s excessive caffeine intake and electrolyte disturbance, alongside his extensive psychiatric history, made it difficult to solely attribute his initial presentation to serotonin hyperexcitability alone, with acute dehydration and stress as possible contributors. Additionally, the patient’s initial presentation did not show any manifestations of neuromuscular excitability, which would be inconsistent with the triad of symptoms used to identify serotonin syndrome. The early presentation of his case would not qualify as serotonin syndrome under the Hunter Criteria, which places major diagnostic significance on the presence of clonus and hyperreflexia to make the diagnosis [1].

This patient did not exhibit neuromuscular excitability until over 24 hours after his arrival at the emergency department. He was also exhibiting muscle rigidity and stiffness, which masked the clonus and hyperreflexia that the Hunter Criteria specifies for diagnosis. The clinical diagnosis and appropriate treatment were delayed until this point. This case also exemplified the importance of collecting collateral information, gathering a detailed history, and careful monitoring upon presentation and admission, which may have aided in diagnosis even when the early presentation of serotonin syndrome appeared incomplete. Although this case was mild, if serotonin syndrome had been recognized sooner, the patient would have received cost-effective, prompt treatment, potentially reducing his length of stay.

## Conclusions

Clear, conclusive diagnostic criteria to identify mild cases of serotonin syndrome are few and far between. Criteria should cast a wider net over mild cases so they do not get overlooked, especially since serotonin syndrome is a potentially life-threatening condition. As more patients are being prescribed serotonergic medications, caution should also be exercised when prescribing medications that may have additional serotonergic activity. As demonstrated by this case, patient education is extremely vital in these cases, to ensure that medications are taken appropriately and to prevent this complication from occurring. Mild cases of serotonin syndrome should continue to be reported in order to help develop updated diagnostic criteria and familiarize more providers with its complex presentation. Quick recognition of serotonin syndrome will allow for appropriate treatment and comprehensive care while also improving patient outcomes, translating into reduced morbidity and mortality.
